# Mattering perception, work engagement and its relation to burnout amongst nurses during coronavirus outbreak

**DOI:** 10.1002/nop2.1075

**Published:** 2021-09-28

**Authors:** Salwa Ahmed Mohamed, Abdelaziz Hendy, Omaima Ezzat Mahmoud, Sayeda Mohamed Mohamed

**Affiliations:** ^1^ Nursing Administration Faculty of Nursing Beni_Suef_ University Cairo Egypt; ^2^ Pediatric Nursing Faculty of Nursing Ain Shams University Cairo Egypt; ^3^ Psychiatric Mental Health Nursing Faculty of Nursing Beni_Suef_ University Cairo Egypt; ^4^ Psychiatric Mental Health Nursing Faculty of Nursing Cairo University Cairo Egypt

**Keywords:** burnout, COVID‐19, mattering, nurses, work engagement

## Abstract

**Aim:**

To assess the mattering perception, feelings of burnout and work engagement amongst nurses during coronavirus outbreak.

**Design:**

Cross‐sectional research design.

**Methods:**

It conducted at Zagazig fever hospital and chest hospital on 280 nurses. A self‐administered questionnaire containing four parts; characteristics, mattering at Work Scale, Burnout scale and Engagement scale.

**Results:**

The present study reported that more than half of studied nurses had moderate mattering level and more than one‐quarter of them had low mattering. More than two‐fifth of studied nurses had moderate level and slight less than one‐third of them had low engagement. More than two‐fifth of studied nurses had moderate level of burnout, whilst slight less than one‐third of them had high burnout, and one‐quarter of them had low burnout.

## 1 INTRODUCTION

Coronavirus is a communicable disease that originates from a large family of viruses that causes the diseases. It is also complicated by the fact that patients may have irregular symptoms. This means COVID‐19 exhibits clinical presentations ranging from asymptomatic to acute respiratory distress syndrome and multiple‐organ failure that leading to death. In addition, COVID‐19 also is transmitted from person to person through respiratory droplets (World Health Organization, 2020).

It is problematic to imagine a scenario, where healthcare worker perceive their work as unimportant to hospitals but are still motivated, satisfied and increase the value of their jobs and hospitals. Therefore, the importance of their activities in the workplace is a major concern for nurses, directors and leadership/head nurses (Poku et al., 2020; Ribeiro et al., 2020). Nurses are employees who are directly affected by the novel coronavirus (Kang et al., 2020). This has affected the psychological abilities of nurses, especially staff working in coronavirus treatment.

The work of nurses includes some specific requirements that make this group particularly vulnerable to emissions, distort perceptions and affect participation in the work. The current situation is much more dangerous, for example in a pandemic situation initiated by COVID‐19 where work demands are very widespread. The fierce tragedy of the current epidemic, the risk of openness of infectious diseases, the expansion of immediate challenges, the lack of personal defences and other clinical supplies, the lack of testing, the limited choice of treatment, the concerns about pollution and to friends and family. Really focussed, honest, moral and complete assets are included in the designated selection (Pfefferbaum & North, 2020).

Opportunities at work include a decrease in social relevance and a diminished ability to set in the workplace, an increase in physiological distress and cardiovascular problems, and a higher frequency of psychological illnesses such as sadness and pressure. Stress and its complex psychosocial risks can also affect other parts of the job, such as job performance, inspiration and work dedication. In the same context, legitimate management of psychosocial risks helps to prevent accidents and non‐participation, increase efficiency and promote prosperity in the work environment (Buerhaus et al., 2020, and the World Health Organization, 2020).

According to much literature, emissions due to healthcare worker relationships in units of work are most prominent in the interaction between caregivers and interested subjects (Guixia & Hui, 2020). Burnout begins with a few symptoms, which increase over time without any intervention. These symptoms are manifested by depersonalization, emotional fatigue and poor nurse performance (Martinelli et al., 2020). They occur in improper work, design, management, organization and poor social work environments and cause physical, psychological and social consequences such as work‐related stress, depression and excretion. (Giménez‐Espert et al., 2020).

According to the burnout of nurses, it also affected nurse performance. Some of the burnout nurses are nurses treating emergency patients (Asih & Trisni, 2015). Emergency nurses are nurses with the ability to give nursing care in the emergency room to gradually or suddenly resolve a patient's problems (Hardiyono et al., 2020).

Mattering perception refers to the psychosocial composition that defines material perception and the feeling that people are important in the world and makes a difference in the lives of others. Nurses have greater awareness of the problem than have high self‐esteem. Increases workplace satisfaction, reduces emissions and improves psychological and overall health. He also points out a positive link between problems and psychological health and participation in the workplace. Negative results are displayed when people feel that they are not important (Efendi et al., 2019; Tong, 2018).

A positive perception of work experience and work environment can have a positive effect on engagement and professional efforts in tasks that cause low stress levels. On the other hand, negative perception of high pressure can lead to resource depletion, that is, fatigue (Permarupan et al., 2020). Previous studies have shown that predictors of burnout include age, gender, marital status, income, education level, years of service, occupation, movement patterns, number of patients assigned to nurses and placement (Alenezi et al., 2019).

Work engagement: It presents three measurements. (i) Commitment characterized by a sense of significance, motivation, challenges, excitement and self‐esteem; (ii) vitality, high levels of energy and mental patience in the workplace, passion and difficulty in investing energy in work. It features confidence to overcome. And (iii) absorption is characterized by experiencing the problem of immersing yourself in your work and abandoning it too deeply and too early (Giménez‐Espert et al., 2020).

According to Hanafi & Reny Yuniasanti (2012), nurses are also often faced with the demands of their own professional code of ethics with those around them trying to save their lives and lives. Besides, we still have to face different kinds of problems, both in our patients and our colleagues. This makes it very easy for nurses to feel much stressed, as these situations can burden them over time. Excessive stress generally adversely affects the individual in coping with the environment, resulting in poor performance, indirect effects on the organization in which the individual works (Hanafi & Reny Yuniasanti, 2012).

Nurses are the main staff of medical institutions. Nurses' interventions in the workplace directly improve the quality of care given to patients and reinforce patient problems in the organization (Pérez‐Fuentes et al., 2019). So far, job engagement has received great attention, has a very good effect on job performance and negatively affects turnover and burnout. Work participation refers to a positive mental state related to work characterized by a sense of commitment and energizing absorption (García‐Sierra et al., 2017; Torabinia et al., 2017).

### 1.1 Significance of the study

Coronavirus pandemic is a genuine well‐being crisis that has influenced nations everywhere on the world. Well‐being crises are a basic psychosocial hazard factor for medical caretakers. As a rule, psychosocial hazards comprise difficult issues as they sway labourers' well‐being, mattering perception, work engagement and burnout. Alongside the effect that a pandemic can have all alone, a key component is the pandemic's insight by the individuals who live with it, particularly bleeding edge labourers, the medical caretakers. Their impression of the estimates taken, the assets accessible, and the pandemic's effect on their work and lives can influence and be influenced by biopsychosocial chances and their outcomes (Liu et al., 2020).

Indeed, there is restricted data with respect to attendants' insights, work commitment and burnout during COVID‐19 flare‐up. Consequently, it is critical to comprehend what medical caretakers think about the infection, and their view of pandemic difficulties and sickness counteraction.

Accordingly, the investigation introduced here means to fill this hole in the information by offering a first way to deal with the impression of COVID‐19 by nursing experts and its relationship with work commitment and burnout during the pandemic.

Finally, this results of the study could give well‐being specialists data to empower them to focus on preparing and different exercises focussed on viably improving medical attendants' insight and improve work commitment and consequently the lessen burnout of their consideration conveyance. The point of this examination is to survey the medical caretakers' impression of making a difference, sensations of burnout and work commitment during the COVID‐19 outbreak.

### 1.2 Aim of the study

This study aimed to assess the mattering perceptions, feelings of burnout, and work engagement during the COVID‐19 outbreak.

### 1.3 Research questions

- What are the levels of nurses' perceptions of mattering, burnout and work engagement?
- Is there relationship between nurses' mattering, work engagement and burnout?

## 2 METHODS

### 2.1 Research design

A cross‐sectional descriptive design was used in this study.

### 2.2 Setting

The study was carried out at Zagazig fever hospital and Zagazig chest hospital, Egypt. Zagazig fever hospital consists of three buildings; the main building consists of five floors containing the inpatient unit and the intensive care unit. The second building consists of a single floor containing outpatient clinics and an emergency unit. The third building consists of a single floor containing the administrative offices. The total nursing staff is 460 nurses working three shifts in inpatient units, intensive care and outpatient clinics. Zagazig Chest Hospital consists of three buildings; the main building includes inpatient units and intensive care unit. The second building consists of one floor with outpatient clinics and an emergency room. The third building consists of a single floor containing the administrative offices. The total number of nurses is 350 nurses with three shifts in inpatient units, intensive care units and outpatient clinics.

### 2.3 Sample

Convenient sample consisted of 280 nurses working in the above mentioned settings and providing care for patients with corona virus.

Sample Size: The estimated sample size was 280 nurses arranged as mentioned below, at confidence level 95%, and the precision rate at 0.05 by using the equation devised by Suresh and Chandrashekara (2012) as the total number of available nurses is 810.

The eligibility criteria for participants were as follows.

#### 2.3.1 Inclusion criteria

‐ Those who have worked as nurses in hospitals for 3 years,
‐ Be actively working during the moment of assessment.

### 2.4 Tool

Four scales were used in the current study:

- Personal data sheet was constructed by the researchers. It includes information about the participants such as age, gender, marital status, Primary specialty, education level, experience and residence.
- Mattering at Work Scale was adapted from (Jung & Heppner, 2017). It was used to measure mattering of nurses at work dependent on the 10‐item divided on two domains as Societal mattering that include 5‐items as I think that society values the work, I do, I feel my work meets a societal need and interpersonal mattering as my co‐worker appreciate my support and help and my co‐worker value my ideas and suggestions. All items were answered using a 5‐point Likert scale format ranging from disagree very much (i), disagree (ii), somewhat (iii), agree (iv), to agree very much (v). Higher scores indicate higher perceptions of mattering at work. Total score ranged from 10–50 score and categorized on three cut points; low 10–23, moderate if score 24–37, high if score 38–50. It was translated into Arabic by researchers. Three experts checked the content validity of these questionnaires in the field of mental health nursing, nursing administration and statistics.
- Burnout scale was adopted from İlhan and et al. (2008). It was used to measure burnout of nurses at work. It consisted of 10‐item, such as I am the person I always wanted to be, I have beliefs that sustain me and I am a very caring person. The tool is on a 5‐point Likert scale, ranging from 1 (never) and 5 (very often) for positive items and reverse‐coded with negative items. High scores indicated higher experiences of burnout. Total score ranged from 10–50 score and categorized on three cut points; low 10–23, moderate if score 24–37, high if score 38–50.
- Work Engagement scale was adopted from Mills and et al. (2012). It was used to measure the nurses' engagement at work. It consisted of 18‐item. It was categorized under three domains as physical engagement include (six items) as I work with intensity on my job and I exert my full effort to my job, emotional engagement as I am enthusiastic in my job and I feel energetic at my job, and cognitive engagement as at work, my mind is focussed on my job at work and I pay a lot of attention to my job. The tool is on a 5‐point Likert scale, ranging from 5 (strongly agree) to 1 (strongly disagree). The total score is ranging from 18–90. High scores indicate higher perceived engagement at work. The levels of work engagement categorized on three cut points; low 18–42, moderate if score 43–67, high if score 68–90.

### 2.5 Pilot study

The pilot study was conducted with 28 nurses who represent 10% of nurses at the previously mentioned settings in order to test the applicability of the constructed tools and the clarity of the included tools. The pilot also served to estimate the time needed for each subject to fill in the questionnaire. A group of experts in the nursing administration departments ascertained the content's validity; their opinions were elicited about the format, layout, consistency, accuracy and relevancy of the tools. Reliability testing was carried out to test the reliability in terms of Cronbach's Alpha for mattering scale = 0.799, burnout scale = 0.837 and Nursing engagement scale = 0.821.

### 2.6 Procedure

Official approval was obtained from Directorate of Health in Zagazig Governorate and the directors of Zagazig fever hospital and Zagazig chest hospital and also from head of different departments at the two hospitals to access the potential participants and the researchers approached the available sample to identify the eligible participants for the current study. The researchers started to contact nurses who met the inclusion criteria of the study. Then, the researchers interviewed all nurses who met inclusion criteria and agreed to participate in the study. The aim of the study was explained to the selected nurses. Written consent was obtained from the study participants. The researchers began data collection by introducing themselves to the participants and explained the content of the study tools to establish initial rapport and gain cooperation between nurses and researchers. All questions related to the study tools were answered, and a detail explanation. Data was collected over a period from March 2020‐June 2020.

### 2.7 Ethical considerations

Official approval for the study was obtained from the scientific research ethics committee of the Faculty of Nursing at Zagazig University (January, 2020), Directorate of Health in Zagazig Governorate and relevant hospital authorities. The study purpose was explained to chairperson of the continuing education department and nurses' supervisors to obtain their support for conducting the study and facilitate the data collection process. The participants were informed that they had the right to withdraw from the study at any time without any adverse consequences. Code numbers were generated and used to ensure patient data confidentiality. Subjects who were willing to participate were asked to sign the informed consent.

### 2.8 Statistical design

Statistics was analysed using the Statistical Package for the Social Sciences (SPSS), version 22. Data were presented using descriptive statistics in the form of Frequency and percentages were used for numerical data, and mean and standard deviation. For parametric analysis, Pearson's correlation coefficient is the test statistics that measures the statistical relationship, or association, between two continuous variables (engagement, mattering and burnout variable). A linear regression model was used to assess the relationship between a scalar response and one or more explanatory variables. The level of statistical significance was set at *p* < .05.

## 3 RESULTS

Table 1 reveals that, 46.40% of nurses were in the age (35 ≤ 44 years old), 87.5% of nurses were female and 76.8% of them were married. Meanwhile, detected that mean of years' experience was 14.4 (5.99) years and 57.1% of them from rural area. As regards the type of residence, 57.1% of nurses were stayed in rural area, whilst 42.9% of nurses were stayed in Urban. About to education level, shows that 60% of nurses had technical health diploma. 52.90% of nurses' work in medical units.

**TABLE 1:** Frequency distribution of personal data amongst nurses r (*N* = 280)

Variables		*N*	%
Age			
25–<35 years old		100	35.70
35–<45 years old		130	46.40
45 or more		50	17.90
Gender			
Male		35	12.5
Female		245	87.5
Marital status			
Married		215	76.8
Not married		65	23.2
Primary specialty			
Medical		148	52.90
Surgical		53	18.90
Critical		79	28.20
Education level			
Diploma		42	15
Technical health institute		168	60
Faculty of nursing		65	23.20
Postgraduate		5	1.80
Years of experience			
1 < 10		95	33.9
10 < 20		105	37.5
20–30		80	28.6
Mean (*SD*)	14.4 (5.99)		
Residence			
Urban		120	42.9
Rural		160	57.1

Table 2 shows that, 53.9% of nurses had moderate societal mattering and 25% of them had low societal mattering with mean score 14.33 ± 2.9. Interpersonal mattering, 50% of studied nurses had moderate level and 27.1% of them had low with mean score 16.21 ± 3.1. Finally, total mattering, 53.2% of studied nurses had moderate level and 28.9% of them had low mattering, with mean score 30.54 ± 6.4.

**TABLE 2:** Frequency distribution of mattering perception amongst nurses (*N* = 280)

	*N*. items	High		Moderate		Low		Mean(*SD*)
		*N*	%	*N*	%	*N*	%	
Societal mattering	5	59	21.1	151	53.9	70	25	14.33 ± 2.9
Interpersonal mattering	5	64	22.9	140	50	76	27.1	16.21 ± 3.1
Total score	10	50	17.9	149	53.2	81	28.9	30.54 ± 6.4

Table 3 reveals that, 45.7% of studied nurses had moderate physical engagement and 28.6% of them had low physical engagement with mean score 19.70 ± 4.01. According, emotional engagement, 47.5% of studied nurses had moderate level and 32.1% of them had low with mean score 16.03 ± 3.35. Cognitive engagement, 42.5% of studied nurses had moderate level and 28.9% of them had low level with mean score 20.11 ± 5.34. Finally, related total engagement, 42.9% of studied nurses had moderate level and 32.1% of them had low engagement, with mean score 55.84 ± 10.17.

**TABLE 3:** Frequency distribution of work engagement domains amongst nurses (*N* = 280)

	*N*. items	High		Moderate		Low		Mean(*SD*)
		*N*	%	*N*	%	*N*	%	
Physical engagement	6	72	25.7	128	45.7	80	28.6	19.70 ± 4.01
Emotional engagement	6	57	20.4	133	47.5	90	32.1	16.03 ± 3.35
Cognitive engagement	6	80	28.6	119	42.5	81	28.9	20.11 ± 5.34
Total	18	70	25	120	42.9	90	32.1	55.84 ± 10.17

**FIGURE 1:**
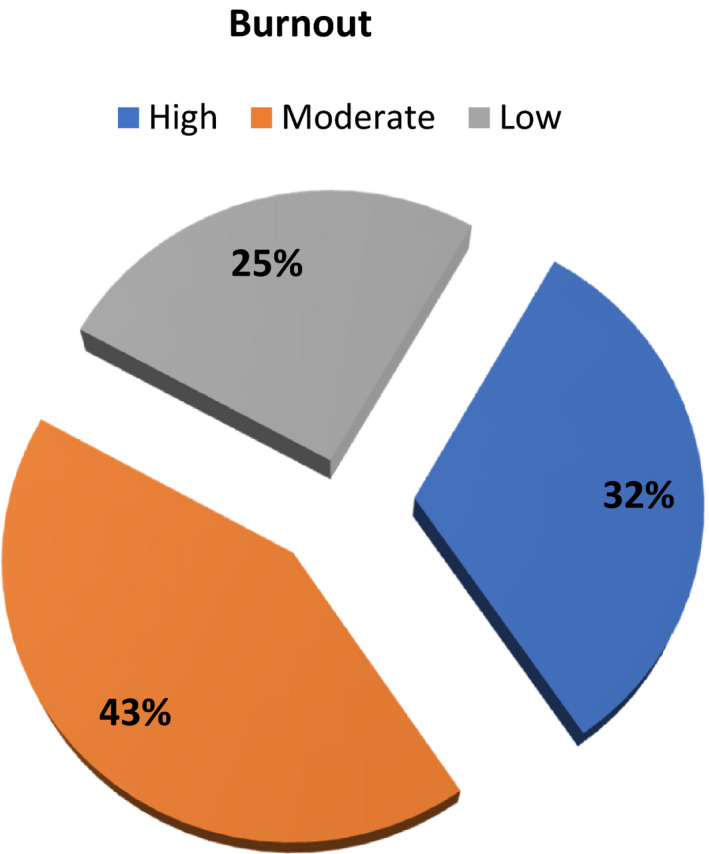
Frequency distribution of burning out level of nurses (*N* = 280)

Figure 1: presents that, 43% of studied nurses had moderate level of burnout, whilst 32% of them had high burnout and 25% of them had low burnout.

Table 4 stated that there was high positive correlation between engagement and mattering at *p*‐value <.01. Whilst, there was high negative correlation between burnout with engagement and mattering at *p*‐value <.01.

**TABLE 4:** Correlation matrix between mattering perception, work engagement and burning out amongst nurses (*N* = 280)

		Mattering perception	Work engagement	Burning out
Mattering perception	r. *p*		.586 .006**	−.592 .009**
Work Engagement	r. *p*	.586 .006**		−.507 .008**
Burning out	r. *p*	−.502 .009**	−.507 .008**	

** High significance <0.01.

Table 5 regression analysis amongst burnout variable and selected demographics represents that, a statistically high significant level detected through *F* test value was 10.501 with *p*‐value .001. This model explain 47% of the variation in burnout detected through R2 value 0.471. Also, explained that increasing engagement by one unit means decrease burnout with (0.197), whilst showed that increasing mattering by one unit means decrease burnout with (0.211), and increased experience by one unit means decrease at burnout with (0.178). But, primary specialty “critical units” had positive effect on burnout.

**TABLE 5:** Linear regressions model for burning out amongst nurses (*N* = 280)

Items	Unstandardized coefficient		Standardized coefficient	*t* test	*p*‐value
	B	Std. Error			
Primary specialty	0.235	0.209	0.189	5.017	.011*
Experience	−0.178	0.311	0.246	7.894	.007**
Mattering	−0.211	0.187	0.199	6.102	.009**
Engagement	−0.197	0.200	0.178	6.944	.008**
R square .471		Model ANOVA 10.501		*p*‐value .001**	

## 4 DISCUSSION

After analysing the collected data, related with first research question, we demonstrated that, more than half (53.2%) of studied nurses had moderate mattering level and more than one‐quarter (28.9%) of them had low mattering. Total engagement, more than two fifth (42.9%) of studied nurses had moderate level and slight less than one‐third (32.1%) of them had low engagement. About to burnout, more than two fifth of studied nurses had moderate level of burnout, whilst slight less than one‐third (32%) of them had high burnout and one‐quarter (25%) of them had low burning out. These results may due to the period in which the data were collected and the study was conducted was a critical time because it was immediately following the first wave of COVID‐19, especially since the hospitals in which the study was held were a fever hospital and another a chest hospital, meaning that they were directly related to the pandemic, so had negative effect on mattering and engagement, also increased burnout feeling.

These results supported with the study conducted by Dadfar et al. (2021) who reported that, the university staff had lower mattering scores. Also, Stoner, 2016 who detected that studied nurses perceive lower levels of mattering to their residents and increased feelings of burnout. Sloan, 2020 at his doctor of nursing practice project revealed that nurses' engagement level was low at pre intervention, whilst improved with 10% postintervention. Awad and Ashour (2020) who stated that, main finding was moderate work engagement amongst nurses. And, cohort with the study performed by Carthon and et al. (2019) who revealed that, only one fifth of studied nurses reported higher levels of engagement. Likewise, Khasne and et al. (2020) who stated that prevalence of personal burnout was 44.6% (903), work‐related burnout was only 26.9% (544). Also, Orozco et al., 2019 who consistent with the present results and revealed that, all subjects who responded to the survey displayed average levels of burnout.

These results similar with the study by Eseadi and Diale (2020) who detected that, nurses reported having high burnout (3.85 ± 0.05). On other hand, Swamy et al. (2020) disagreement with current results because showed that, one‐third of studied nurses reported burnout. Orgambídez‐Ramos et al. (2017) stated that, the lack of economic resources and personnel in hospitals and the greater demands at work make nurses particularly susceptible to experiencing burnout in the work context. Effective interventions and strategies are required to reduce burnout amongst nurses (Hailay et al., 2020).

As regards to the correlation between engagement and mattering amongst nurses there was high positive correlation between engagement and mattering. Whilst, there was high negative correlation between burnout with engagement and mattering. Also, linear regression model explained that engagement, mattering, increase at experience caused decreasing burnout, whilst worked at critical units caused increased at nurses' burnout. This finding could be interpreted as nurses during pandemic suffer from psychological stress related to work load that affect their engagement and mattering perception. Increasing work engagement and improve mattering perception amongst nurses lead to low feeling of burnout.

These results are consistent the findings of Flett & Zangeneh, 2020 who stated that, the vital role of mattering was decreasing feelings of loneliness, safeguarding the mental health and improve engagement at all times but especially when in crisis situations. Also, supported with the study conducted by Haizlip et al., 2020 who reported that, higher levels of mattering at work were associated with lower burnout and higher engagement. Epstein and et al. (2020) who mentioned that, mattering, moral distress and secondary traumatic stress as they relate to burnout. Liu, and Aungsuroch (2019) reported that, both work stress and the perceived social support as mattering moderately and directly affected burnout. And, supported with the study by Dempsey, and Assi (2018) who reported that, the vital connection of nurse engagement to the experience of care, and ultimately to nurse and patient outcomes, is clear. Also regular with Park and Kim (2020) who demonstrated that, self‐esteem, compassion satisfaction, and secondary traumatic stress were also statistically significant factors affecting burnout.

### 4.1 Limitation of the study

There was no specific method for data collection from nurses during COVID‐19 outbreak. So the researchers identified the most suitable methods as Google form questionnaires to data collection to prevent spread COVID‐19 infection.

## 5 CONCLUSION

Based on the findings of the present study, the study concluded that, nurses had moderate mattering perception level. Related total work engagement, nurses had moderate level. According burnout, nurses had moderate level of burning out level. Also, there was a statistically significant positive correlation between engagement and mattering perception. Whilst, there were a statistically significant negative correlation between burning out with engagement and mattering.

### 5.1 Recommendations

The current study was limited to nurses working at a two central hospital, therefore, recommended that a larger sample size be used in the future studies to enhance external validity.

Future researches can emphasis on the causal associations between nurses' characteristics and mattering and engagement. Training workshop for nurses about coping mechanism for burnout feeling.

Design training programme for head nurses and directors for enhancing engagement and mattering of nurses.

Development of Psycho‐educational nursing programmes should be held to help the nurses to deal with biopsychosocial risks faced them during COVID−19 (second wave of pandemic).

## CONFLICT OF INTEREST

No conflict of interest has been declared by the authors.

## AUTHOR CONTRIBUTIONS

All authors contributed to conception and study design, data collection, analysis, interpretation, manuscript writing, reviewing and revising it. All authors read and approved the final manuscript.

## Data Availability

All data are already present at the published article.
